# Brassinolide and BZR1 are up-regulated in a parthenocarpic mutant of prickly pear

**DOI:** 10.1007/s00299-025-03514-w

**Published:** 2025-05-23

**Authors:** Rameshkumar Ramakrishnan, Udi Zurgil, Danuše Tarkowská, Ondřej Novák, Miroslav Strnad, Noemi Tel-Zur, Yaron Sitrit

**Affiliations:** 1 French Associates Institute for Agriculture and Biotechnology of Drylands, The Jacob Blaustein Institutes for Desert Research, Ben–Gurion University of the Negev, Sede-Boqer Campus, 84990 Negev, Israel; 2 Laboratory of Growth Regulators, Faculty of Sciences, Palacký University and Institute of Experimental Botany AS CR, Šlechtitelů 27, 783 71 Olomouc, Czech Republic; 3 Katif Research Center for R&D, Ministry of Innovation, Science and Technology, 8771002 Netivot, Israel; 4 The Jacob Blaustein Institutes for Desert Research, Ben-Gurion University of the Negev, Sede Boqer Campus, 84990 Netivot, Israel

**Keywords:** *Opuntia ficus-indica*, Brassinolide, Brassinosteroids, BZR1, Cacti, Fruit, Ovule, Parthenocarpy, Prickly pear

## Abstract

**Key message:**

Parthenocarpic fruit development in prickly pear involves up-regulation of the transcription factor BZR1 and increased levels of brassinolide in developing ovules.

**Abstract:**

We explored the complex process of parthenocarpic fruit development in prickly pear *Opuntia ficus-indica* (Cactaceae) by comparing the fruits of the parthenocarpic Beer Sheva1 (BS1) mutant and revertant non-parthenocarpic fruits. The mutant plants produce flowers with enlarged ovules that develop into degenerated seed-like stony structures. Pollen tubes fail to penetrate the ovule, resulting in the formation of lignified and hard seed coat brown in colour. Some new stems on BS1 plants bear normal revertant flowers containing small and viable fertilized ovules. BS1 thus provides a unique model for elucidating the regulatory mechanisms underlying parthenocarpy in prickly pear. Our working hypothesis was that parthenocarpy is induced by elevated levels of brassinolide in the ovules of BS1. By comparing transcriptomes, we identified 7717 differentially expressed genes between BS1 and the revertant among them brassinosteroid-related genes. Quantification of the brassinosteroids confirmed higher brassinolide levels and up-regulation of the brassinosteroid positive regulator BRASSINAZOLE RESISTANT1 (BZR1) in BS1 ovules compared to revertant ovules displaying normal seed development. Thereby, implicating the involvement of brassinolide in ovule development, fruit phenology, and parthenocarpy. The early flowering and fruit ripening observed in BS1 support our hypothesis that brassinolide promotes parthenocarpic fruit development and ripening.

**Supplementary Information:**

The online version contains supplementary material available at 10.1007/s00299-025-03514-w.

## Introduction

Fruit development, initiated primarily by pollen fertilization of the ovule and the central cell, is followed by embryogenesis, endosperm development, and seed maturation. However, in some fruit species, such as apples, bananas, cucumbers (Li et al. 2014), tomatoes (Gupta et al. 2021), citrus (Mesejo et al. 2016), and prickly pear *Opuntia ficus-indica* (Cactaceae)—the fucus species of this research—plant hormonal imbalance may result in the production of fruits without seeds, a trait known as parthenocarpy. Parthenocarpic fruits often display elevated levels of hormones compared to their seed containing counterparts, underscoring significant hormonal influence on the initial stages of fruit set (Li et al. 2014). The plant hormones intimately linked to parthenocarpy include cytokinins, auxins, gibberellins (GAs), and brassinosteroids (BRs), all of which play central roles in regulating and enhancing fruit set. GAs and BRs, in particular, share critical roles in promoting hypocotyl elongation, in controlling the timing of flowering and seed germination (Fu et al. 2008; Domagalska et al. 2010; Li et al. 2024). The interplay between these two groups of phytohormones is evident in their mutual influence on gene expression and biosynthesis of the other, demonstrating a sophisticated level of interconnectivity within the plant hormone signaling network (Domagalska et al. 2010; Li et al. 2024). An example of the crucial co-regulation of these hormones in plant development may be found in the severe dwarf phenotype observed in *Arabidopsis thaliana* mutants that lack the ability to synthesize GAs or BRs or to perceive either GA or BR signals (Depuydt and Hardtke 2011).

BRs exert significant growth-promoting effects, contributing to the formation of organ boundaries, the elongation of stems, the differentiation of vascular tissue and stomata, the development of roots, and the germination of seeds (Fu et al. 2008; Domagalska et al. 2010). BRs are also responsible for inducing photomorphogenesis in seedlings, enhancing male fertility, promoting reproductive growth, initiating flowering, driving fruit development, and facilitating plant responses to both biotic and abiotic stresses (Ye et al. 2010; Gendron et al. 2012). The current comprehensive understanding of BR biosynthesis and signaling derives primarily from extensive studies in *A. thaliana* (Tong et al. 2014). Several genetic elements critical to the BR signaling pathway have thus been identified in *A. thaliana*, including BRI1-ASSOCIATED RECEPTOR KINASE1 (BAK1), the receptor-like BRASSINOSTEROID-INSENSITIVE1 (BRI1), BRASSINOSTEROID-SIGNALING KINASE1 (BSK1), the transcription factors BRI1-EMS-SUPPRESSOR1 (BES1) and BRASSINAZOLE RESISTANT1 (BZR1), and the structural gene coding for brassinosteroid-6-oxidase 1 (BR6ox1), a polypeptide involved in the C-6 oxidation of BRs (He et al. 2005; Ye et al. 2011; Wang et al. 2012; Tong et al. 2014; Kour et al. 2021). It has also been shown that the BR signaling mechanism in rice (*Oryza sativa*) is similar to that in *A. thaliana*, with key genetic components, such as OsBRI1, OsBAK1, OsBIN2, and OsBZR1 being crucial to BR biosynthesis (Bai et al. 2007; Tong et al. 2014). Genetic and functional analyses of these components have shown a conserved BR signaling pathway in rice and *A. thaliana*, indicating a shared fundamental, mechanism for BR production across these species (Bai et al. 2007; Tong et al. 2014).

BRs are produced from sterols through a receptor-kinase-mediated plant signaling pathway (Gendron et al. 2012; Li et al. 2024). In this pathway, the receptors BRI1 and BAK1, together with several positive regulators (such as BSK1, BSU1, and PP2 A) and negative regulators (including BKI1, BIN2, and 14–3-3 protein), regulate the activities of the BES1 and BZR1 transcription factors (Ye et al. 2011; Kour et al. 2021). These factors, in turn, modulate the expression of hundreds to thousands of genes involved in various BR responses (Ye et al. 2011; Li et al. 2024). Specifically, BR binds to and activates the receptor kinase BRI1, initiating a signaling cascade that regulates the activity of BES1 and BZR1 (Ye et al. 2011; Kour et al. 2021). BRI1 forms a complex with BAKI1, and when it binds BR, BKI dissociates to the cytosol. This interaction enables auto- or trans-phosphorylation of both BRI1 and BAK1, which activates the transcription factors BZR1 and BES1 (Bai et al. 2007; Ye et al. 2011; Tong et al. 2014; Kour et al. 2021). When the levels of BRs are low, BES1 and BZR1 are phosphorylated by the kinase BIN2 (Bai et al. 2007; Ye et al. 2011; Tong et al. 2014; Kour et al. 2021). Phosphorylated BZR1 and BES1 are then removed from the nucleus by 14–3-3 proteins and retained in the cytoplasm, where they are degraded. Conversely, when BR levels are high, activation of BR signaling kinases (BSKs) and the receptor-like cytoplasmic kinase CDG1 leads to the activation of BSU1 phosphatase, which, in turn, dephosphorylates and hence inactivates BIN2. Inactivation of BIN2 halts the phosphorylation of BZR1 and BES1, preventing their degradation (Ye et al. 2011; Kour et al. 2021). Dephosphorylated BZR1 and BES1 subsequently translocate to the nucleus, where they regulate gene expression in response to BR signaling. This intricate process highlights the pivotal role of BR-BRI1 signaling in regulating plant growth and development (Bai et al. 2007; Ye et al. 2011; Tong et al. 2014; Kour et al. 2021). This regulation controls the activities of the BES1 and BZR1 transcription factors that modulate the expression of genes involved in BR responses (Ye et al. 2011). BES1/BZR1 regulation impacts a variety of biological processes and controls both sexual (ovule, anther, and pollen) and asexual (hypocotyl, stem, fruit, and root) organ development in various crops (Yu et al. 2022). For example, recent studies have highlighted the significant roles of BES1 and BZR1 in tomato fruit development and ripening (Liu et al. 2014; Yu et al. 2022). Overexpression of BZR1 leads to elongated fruit shapes, along with enhanced accumulation of carotenoids and promotion of ripening. Additionally, microscopic analysis revealed a reduction in the number of cell layers in the fruit pericarp of tomato plants with elevated BZR1 levels (Liu et al. 2014; Yu et al. 2022; Li et al. 2024). Finally, mutants lacking BRs or insensitive to BRs exhibit various developmental anomalies, including stunted growth, reduced apical dominance, altered photomorphogenesis under dark conditions, clustered stomata, and male infertility (Bishop 2003).

In contrast to many other plant hormones, BRs appear to be produced and act within the same cell or tissue, rather than undergoing'long-distance'transport to distinct sites of synthesis and action (Symons and Reid 2004; He et al. 2005). Since the activity of BRs commences at the site of synthesis, it is necessary that the biosynthesis of BRs and the signaling cascade be intricately regulated to ensure balanced cell expansion during normal plant growth and development (Symons and Reid 2004; He et al. 2005).

Both naturally occurring and induced parthenocarpy can be leveraged to advance our understanding of the hormonal and genetic mechanisms that drive fruit and seed development (Li et al. 2014; Mesejo et al. 2016). A model plant species that is particularly suitable for this purpose is a spontaneous mutant of the cactus *O. ficus-indica*, known as Beer Sheva1 (BS1), that bears parthenocarpic fruits. This mutant was first found among plants of the commercial cultivar'Ofer'growing in Beer-Sheva, Israel (Weiss et al. 1993). The plant develops flowers with larger ovules than'Ofer', which mature into two types of structure—small, degenerated seed-like structures and large stony seed-like structures. Importantly, approximately 10% of the branches of BS1 plants produce normal flowers that have reverted to their original'Ofer'progenitor phenotype, containing small ovules and setting fruits with viable normal seeds (Weiss et al. 1993). BS1 fruits exhibit several distinctive characteristics, including early fruit ripening, a longer fruit neck, and fewer, smaller spines on the peel, setting them apart from both revertant fruits and fruits of the'Ofer'cultivar. In addition, at anthesis, the ovules of BS1 are five times larger than those found in revertant fruits (Weiss et al. 1993).

The distinct fruit phenotype of BS1 indicates the involvement of GAs in fruit development. Indeed, in a previous study, we found higher levels of active GAs in BS1 ovules compared to revertant ovules (truly isogenic lines), and higher expression of GA-related genes. Crosstalk between BR and GA via BZR1 was first revealed in *A. thaliana.* BZR1 not only controls the expression of many signaling components of other hormonal pathways but also directly co-regulates genes involved in their biosynthesis (Sun et al. 2010). Although much is known about the involvement of brassinosteroids in ovules and fruit development of many species, almost nothing is known about cacti in general and specifically in *O. ficus-indica*. Studying the regulation of seeds development in *O. ficus-indica* is becoming important due to global warming and the potential of this crop. However, the seed number and size are an obstacle for marketing—seedless fruit can be better accepted by consumers.

Taking the above into consideration, we focused, in the current study, on characterizing the level of the BR hormone, brassinolide, and the BR transcription factor, BZR1, during ovule development in the parthenocarpic fruit of *O. ficus-indica*. We quantified the endogenous levels of BRs in the BS1 mutant and revertant ovules, and we compared the transcriptome profiles of BS1 and revertant ovules using RNA-seq, followed by RT-qPCR analysis.

## Materials and methods

### Plant materials and growth conditions

**Fig. 1:**
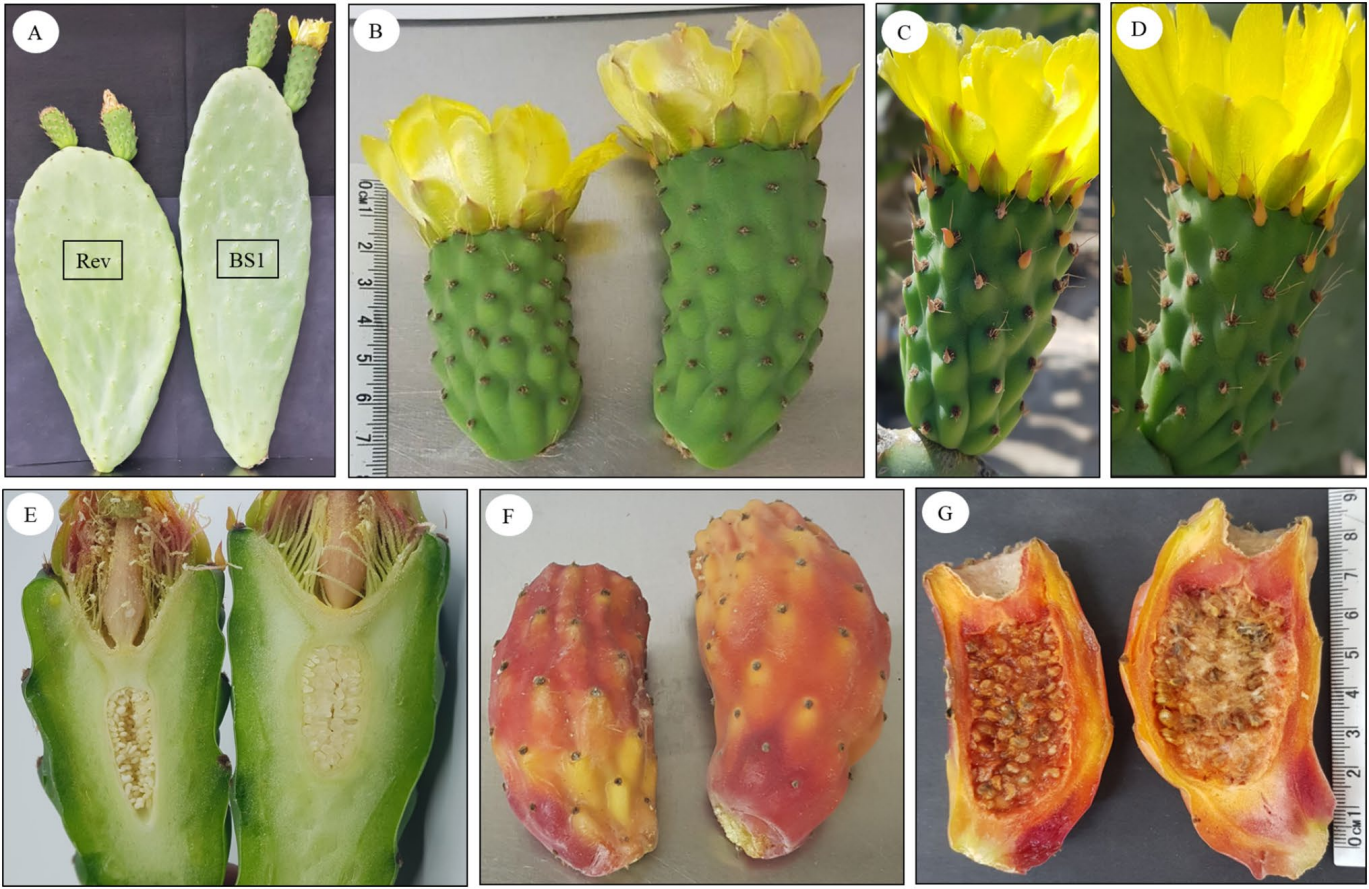
Phenotypes of revertant (left panels) and BS1 (right panels). **A** Morphology of the stems—shorter rounded revertant stem compared to longer slim BS1 stem. **B** Morphology of the flower buds at anthesis—shorter revertant bud compared to BS1 bud. **C**, **D** Morphology of a revertant and BS1 flowers, showing shorter spines on revertant than on BS1. **E** Morphology of revertant and BS1 ovules. **F** Morphology of ripe revertant and BS1 fruit showing long “neck” of the BS1 fruit. **G** Cross-sections of revertant and BS1 fruit showing small viable seeds in the revertant fruit and large degenerated seed-like structures in the BS1 fruit. The ruler presented in B is also for panels C, D, and E (same bud flowers). Ruler in panel G is also for panel F (same fruit)

*O. ficus-indica* BS1 mutant plants were grown on the Bergmann Campus of Ben-Gurion University of the Negev, Beer-Sheva, Israel. Average minimum and maximum temperatures in the summer are 21.6–34.5 °C and 7.7–17.4 °C in the winter. The plants were drip irrigated and fertilized every 3 days in the summer and every 7 days in the winter. The irrigation water contained fertilizer (23 N–3P–20 K) at N concentration of 30–40 mg l^−1^ was applied continuously. For the morphological study, 10 BS1 stems with their flower buds and 10 revertant stems with their flower buds were randomly harvested, with the difference between BS1 and revertant stems being easily discernible from the stem and bud phenotypes (Fig. 1). Stem morphology was assessed in terms of length, diameter, weight, and number of flower buds per stem. Flower buds (pre-anthesis, 5 cm long) and flowers (at anthesis, 7 cm long) were analyzed in terms of diameter, length, and weight. All characteristics in both stems and flower buds were determined within 24 h of harvest. The flower buds were harvested early in the morning; however, BS1 flowers were harvested earlier than revertant, since they flower 2 weeks before.

### Analysis of BRs

BRs and their precursors were determined in ovule samples in six replicates before and at anthesis and frozen tissues were grounded by a mortar and pestle in liquid nitrogen, according to the methodology outlined by Tarkowská et al., (2016). The samples were analyzed using an ultra-high performance liquid chromatography system (Acquity UPLC™; Waters, Milford, MA, USA) coupled to a triple-stage quadrupole mass spectrometer (Xevo® TQ MS; Waters MS Technologies, Manchester, UK) equipped with an electrospray interface (ESI). The LC–MS data were processed and analyzed using MassLynx™ Software (version 4.1, Waters, Manchester, UK). The BRs detected were: campesterol (CR), 6-deoxocathasterone (6-deoxoCT), 6-deoxoteasterone (6-deoxoTE), 3-dehydro-6-deoxoteasterone (3 d-6-deoxoTE), 6-deoxotyphasterol (6-deoxoTY), 6-oxocampestanol (6-oxoCN), cathasterone (CT), teasterone (TE), typhasterol (TY), castasterone (CS), brassinolide (BL), 28-norteasterone (norTE), homocastasterone (homoCS), homobrassinolide (homoBL), 28-norbrassinolide (norBL), 28-norcastasterone (norCS), 24-epicastasterone (epiCS), and 6-deoxo-norcastasterone (6-deoxo-norCS). The BRs that were not detected: homodolicholide, dolichosterone, dolicholide, homodolichosterone, and homobrassinolide.

### RNA extraction and cDNA synthesis

For RNA extraction and BR analyses, ovules were collected from flower buds before anthesis and from flowers at anthesis. The samples were snap frozen in liquid nitrogen, lyophilized, and stored at −80 ºC until use. Total RNA was extracted from the ovule samples. The tissue was transferred to a tube with liquid nitrogen for grinding with three metal beads for 2 min at 25 Hz (Rotator mixer machine). PVP (10 mg) was added to the tissue powder (15–100 mg) and liquid nitrogen. The lysis buffer (Quick-RNA™ Miniprep Kit (Zymo Research, CA, USA) was supplemented with β-mercaptoethanol (2 µl/ml). The samples were vigorously shaken immediately, followed by an incubation at 35 °C for 5 min. Then, genomic DNA was removed using the DNase I kit from Zymo (Thermo Fisher Scientific Inc.; USA).

A NanoDrop 1000 spectrophotometer (Thermo Fisher Scientific Inc.; USA) and 1.0% agarose gel electrophoresis were used to analyze the quantity and quality of the total RNA. Subsequently, 200 ng of purified RNA samples were reverse transcribed into cDNA using the iScript cDNA synthesis kit (BioRad Inc., USA). Briefly, strands were separated at 70 °C for 5 min flowed by cooling and RT activation and elongation, according to the manufacturer’s instructions, i.e., 5 min 25 °C priming, 30 min 46 °C elongation, 1 min 95 °C inactivation, and 4 °C until freezing.

### De novo transcriptome assembly

This part of the study was conducted using an Illumina HiSeq 4000 platform (Illumina, Chicago, USA) with ovule samples collected from BS1 and revertant at anthesis. De novo transcriptome assembly was carried out using SPAdes version 3.10.1 on raw Illumina reads with default parameters and a k-mer value of 55 (Bankevich et al. 2012). Contigs were filtered after assembly to a minimum length of 200 base pairs. Kallisto was used to calculate transcript expression levels (Bray et al. 2016). A sequence search against the SwissProt database using DIAMOND in blastx mode was used to identify genes and open-reading frames (ORFs), followed by KEGG orthology annotation (Buchfink et al. 2015; Zaru and Orchard 2023). The exactTest technique with TMM (trimmed mean of M-values) normalization was used in edgeR for the analysis of the differential transcript abundance (McCarthy et al. 2012; Robinson et al. 2010), with a false discovery rate (FDR) correction using the method of Benjamini and Hochberg (1995). Finally, the RNA-Seq data were visualized and analyzed using IDEP (Ge et al. 2018).

### Expression of differentially expressed genes related to BRs

Transcriptome data were used to determine the expression of differentially expressed genes (DEGs) linked to BR (specifically receptors BRI1 and BAK1, the positive regulator BSK1, the transcription factor BZR1, and the biosynthetic gene BR6ox1. Validation of the transcriptome results for some genes was carried by RT-qPCR as described below.

### Validation of gene expression

Using the transcriptome data, we analyzed ovule samples from both pre-anthesis and anthesis stages of the BS1 and revertant phenotypes for BR-related genes, specifically BRI1, BAK1, BSK1, BZR1, and BR6ox1. Actin was used as the internal reference gene. Primers were created using Primer-BLAST (NCBI). We used the following gene forward/reverse (F/R) primers in our study: BAK1 (F: 5’ CGTGAAATTGTGGTGGCAGC 3’, R: 5’ GGGCAAGGCATCCTCTTTCA 3’), BRI1 (F: 5’ GCCTAGATGGGGCTGACAAG 3’, R: 5’ ACTAGGGCACGAGCAACATC 3’), BZR1 (F: 5’ CGAGCTCAGGGCAACTACAA 3’, R: 5’ TCTCCATCGGAGGTGGCTTA 3’), BSK1 (F: 5’ GGTGGCTGTTGTAGAAGGGTG 3’, R: 5’ GACACTCTGGCCGCATGTAG 3’), and BR6ox1 (F: 5’ AAACAGAGCCGCAAGCAGAG 3’, R: 5’ TCCATCTCAACAAGGCGGTG 3’). All primers were tested using a five-serial dilution calibration method test and were selected only if the efficiency was shown to be 90–110%.

### RT-qPCR analyses

RT-qPCR was conducted with an ABI 7500 real-time PCR System (Applied Biosystems, CA, USA) using the SYBR® Green PCR Master Mix (Applied Biosystems, CA, USA). Ten microliters of reaction mixture contained 2 µL of diluted cDNAs (~ 15 ng/μL), 5 µL of SYBR® GREEN PCR Master Mix, 0.3 µL of each primer (10 µM) and 2.7 µL of doubly distilled water. Gene-specific primers were designed using Primer3web (version 4.1.0). The PCR reaction conditions were as follows: 50 °C for 2 min; 95 °C for 10 min; 40 cycles at 95 °C for 15 s; 56 °C for 30 s; and 72 °C for 40 s. The melting curve was generated by heating the amplicon from 60 to 95 °C to confirm primer specificity. Each PCR reaction was repeated three times with four biological replicates. Relative fold changes in gene expression were calculated using the comparative 2^−ΔΔCT^ method.

### Statistical analysis

Six, four, and three independent biological replicates were used for determining concentrations of BRs, for expression analysis, and for transcriptome analysis, respectively. The data are presented as means ± SE and were analyzed using Student’s t test, followed by Tukey's HSD for post hoc tests (*P* < 0.05). All statistical analyses were carried out using GraphPad Prism software.

## Results

### The BS1 mutation results in phenotypic alterations in stem and floral morphology

The distinctive traits of BS1 vs. revertant stems and flowers (Fig. 1A–F) included elongated and narrower stems (Fig. 1A), longer flowers (Fig. 1B), and longer spines on the flower bud (Fig. 1C, D). In addition, BS1 ovules were larger and less compact than revertant ovules (Fig. 1 E), and ripe parthenocarpic fruits had a longer “neck” than revertant fruits and contained numerous small, degenerated seeds as well as large, hard, lignified, brown unfertilized ovules (Fig. 1F, G).

**Fig. 2:**
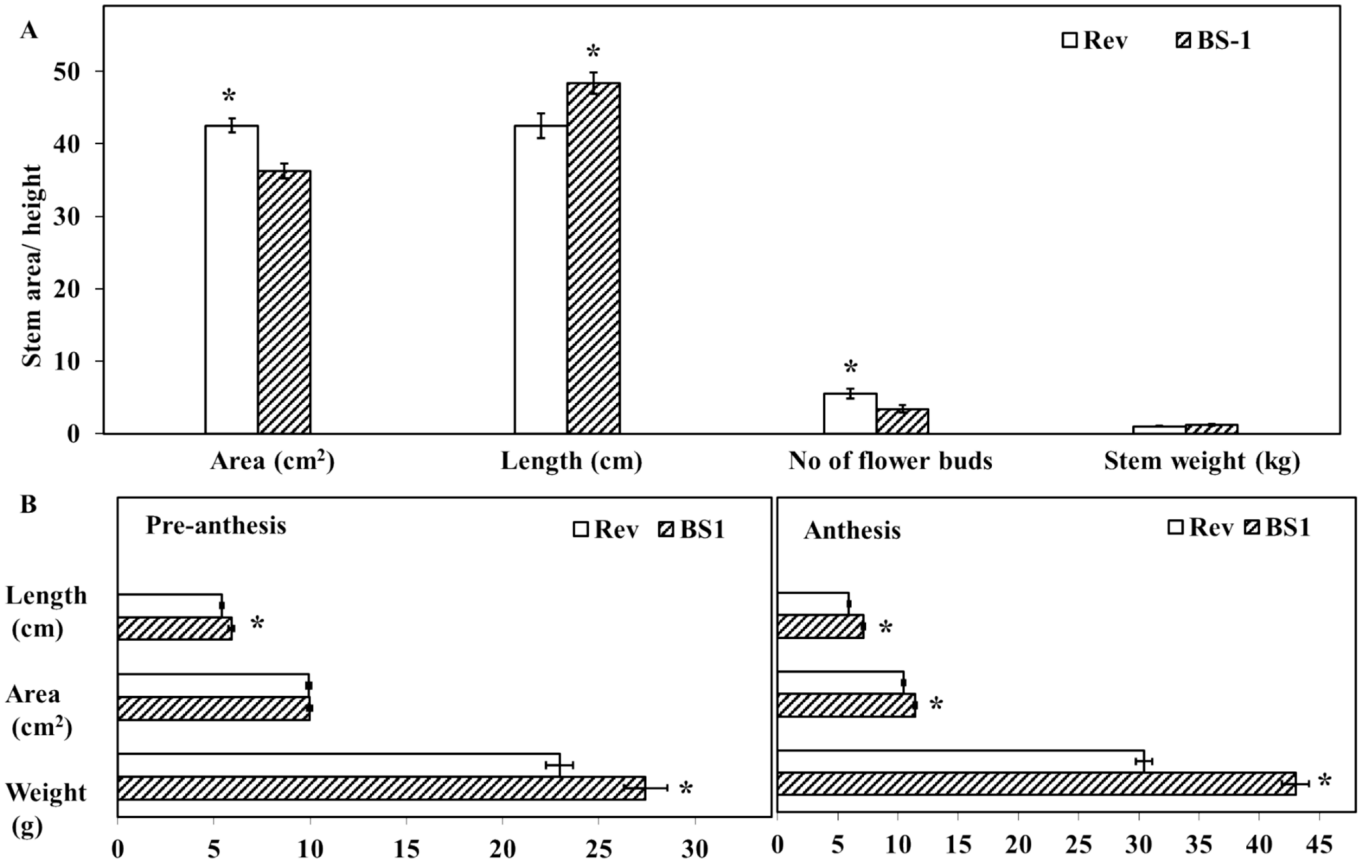
Morphological differences between BS1 and the revertant stems and flower buds. **A** Comparison of morphology in BS1 and revertant stems in terms of length, diameter, number of flower buds per stem, and stem weight. **B** Comparison of morphology of flower buds on BS1 and revertant stems at the pre-anthesis and anthesis stages. Data are presented as means ± SE (*n* = 10) and were analyzed using Student’s t test. Asterisks denote statistically significant differences at *P* < 0.05

A quantitative examination of differences between BS1 and revertant stems and flowers revealed that BS1 stems were significantly longer than revertant stems, but the BS1 stem area was significantly smaller, and the number of buds/flowers was significantly lower (Fig. 2A). Stem weight did not differ significantly between the two types of stem (Fig. 2A). Flower morphological data indicated that BS1 flower weight and length were significantly higher vs. revertant flowers at both the pre-anthesis and anthesis stages (Fig. 2B). These morphological differences in stems and flowers suggest an active mechanism that potentially influences the levels of BRs or BR regulatory elements during fruit development.

### Higher levels of brassinolide are detected in BS1 compared to revertant

**Fig. 3:**
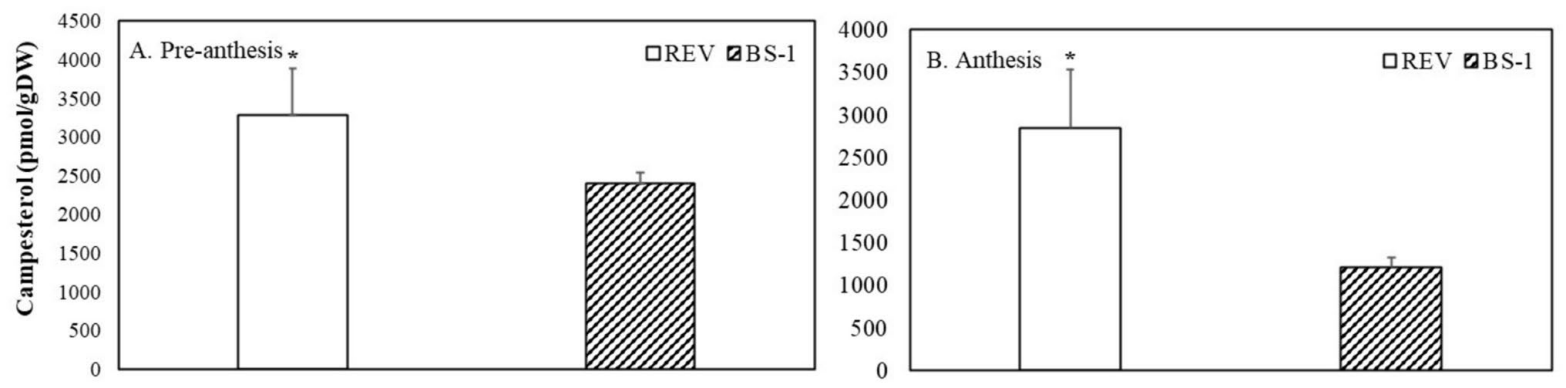
Concentrations of campesterol, the precursor of the brassinosteroids, in ovules of BS1 and revertant flowers **A** during pre-anthesis when the flower buds were 5 cm long and **B** at anthesis when the flowers were 7 cm long. Data are presented as means ± SE (*n* = 6) and were analyzed by Student’s t test. Asterisk denotes a statistically significant difference at *P* < 0.05. Campesterol was converted to campestanol (CN) and then to 6-deoxocastasterone (6-deoxoCS) through the late C-6 oxidation pathway (Fig. 4A, B)

The levels of campesterol in the ovules of the revertant flowers, one of the precursors in the sterol-specific pathway, were 1.3- to 2.3-fold higher, during pre-anthesis and anthesis respectively, than in BS1 ovules (Fig. 3A, B).

**Fig. 4:**
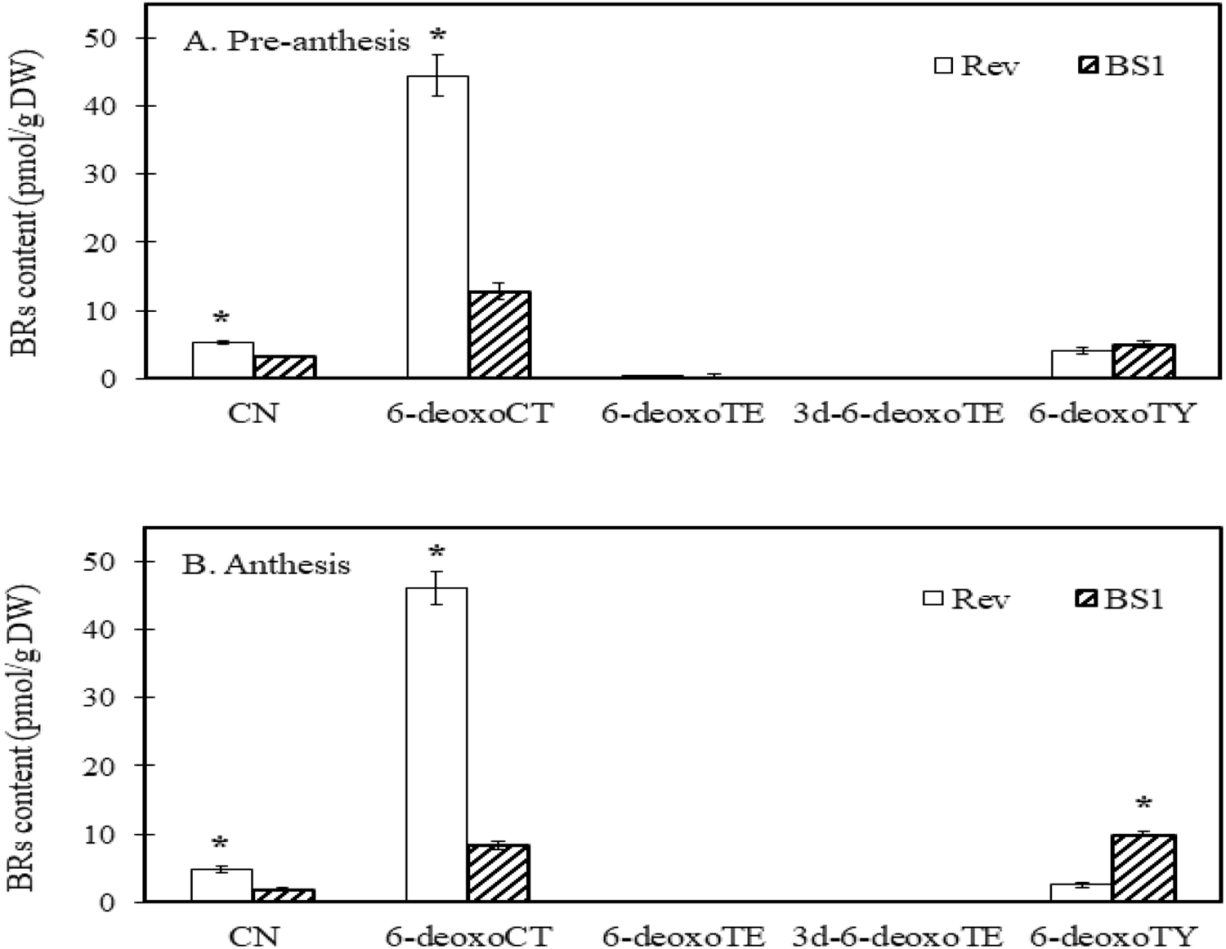
Synthesis of brassinosteroids (BRs) in the late C-6 oxidation pathway: concentrations of campestanol (CN), 6-deoxocathasterone (6-deoxoCT), 6-deoxoteasterone (6-deoxoTE), 3-dehydro-6-deoxoteasterone (3 d-6-deoxoTE), and 6-deoxotyphasterol (6-deoxoTY) during ovule development in BS1 and revertant flower buds. BRs were analyzed in ovules sampled before anthesis and at anthesis when the flower buds were 5 and 7 cm long, respectively. Data are presented as means ± SE (*n* = 6) and were analyzed by Student’s t test. Asterisks denote statistically significant differences at *P* < 0.05

**Fig. 5:**
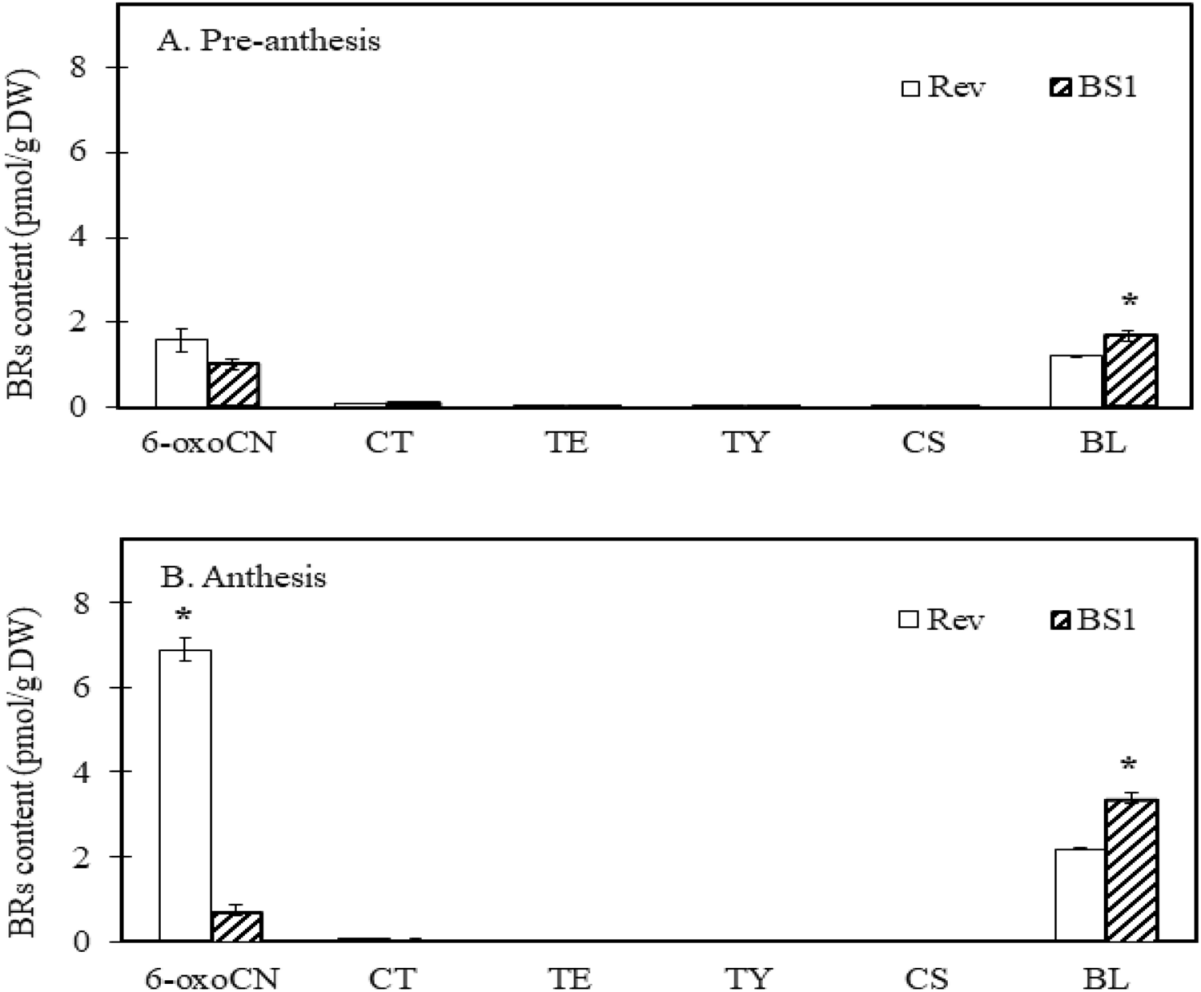
Synthesis of brassinosteroids (BRs) in the early C-6 oxidation pathway: concentrations of 6-oxocampestanol (6-oxoCN), cathasterone (CT), teasterone (TE), typhasterol (TY), castasterone (CS), and brassinolide (BL) in BS1 and revertant ovules. BRs were analyzed in ovules sampled before anthesis and at anthesis when the flower buds were 5 and 7 cm long, respectively. Data are presented as means ± SE (*n* = 6) and were analyzed by Student’s t test. Asterisks denote a statistically significant difference at *P* < 0.05

Remarkably, the concentrations of CN and 6-deoxocathasterone (6-deoxoCT) in the revertant ovules at both pre-anthesis and anthesis were significantly higher than those in BS1 ovules (Fig. 4A, B). Conversely, the level of 6-deoxotyphasterol (6-deoxoTY) was higher in BS1 ovules at anthesis (Fig. 4B), while brassinolide, the active brassinosteroid derived from the BR-specific early C-6 oxidation pathway, was notably elevated in BS1 ovules compared to revertant ovules during both pre-anthesis and anthesis (Fig. 5A, B).

**Fig. 6:**
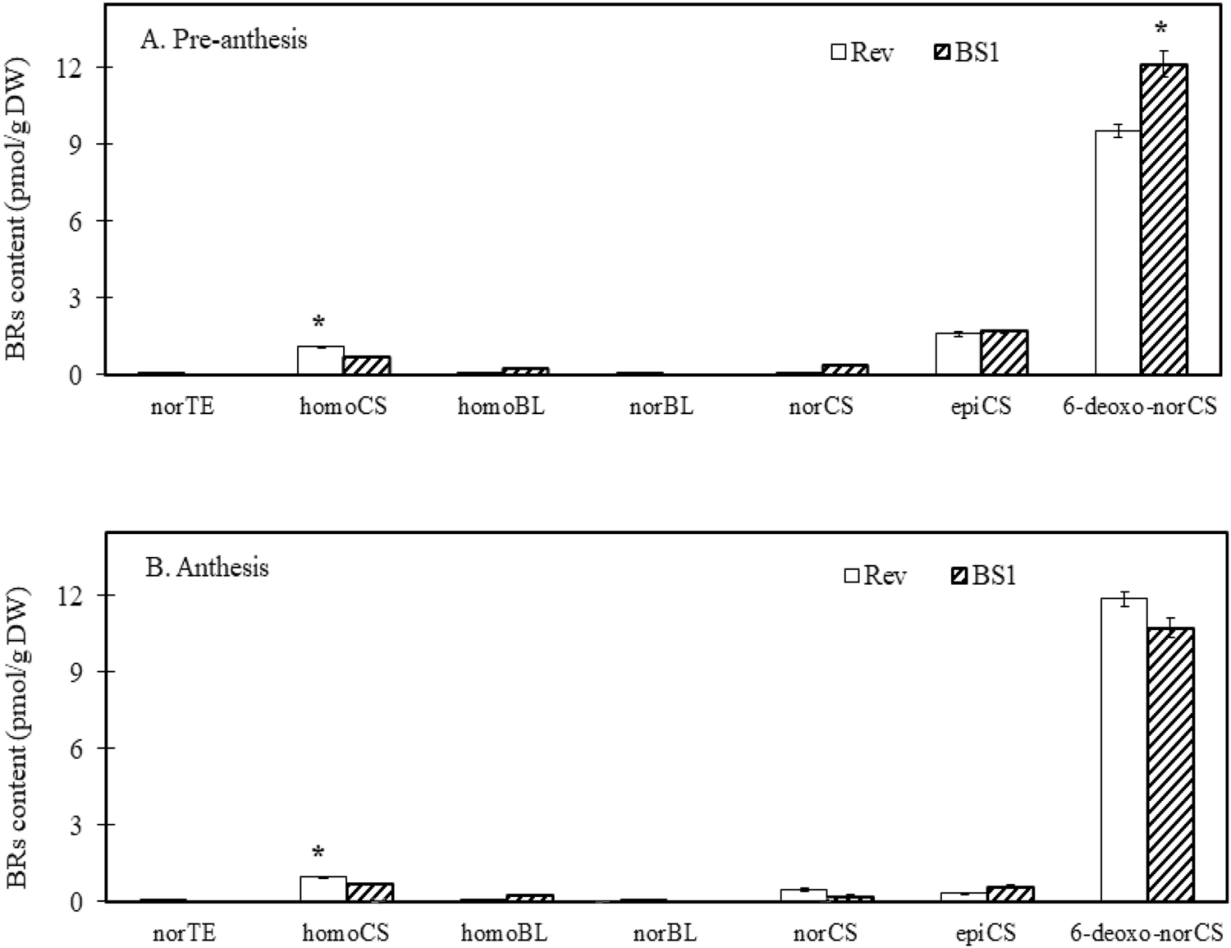
Comparison of the concentrations of various brassinosteroids (BRs) in BS1 and revertant ovules: 28-norteasterone (norTE), homocastasterone (homoCS), homobrassinolide (homoBL), 28-norbrassinolide (norBL), 28-norcastasterone (norCS), 24-epicastasterone (epiCS), and 6-deoxo-norcastasterone (6-deoxo-norCS). BRs were analyzed in ovules sampled before anthesis and at anthesis when the flower buds were 5 and 7 cm long, respectively. Data are presented as means ± SE (*n* = 6) and were analyzed by Student’s t test. Asterisks denote a statistically significant difference at *P* < 0.05

No significant differences were observed between BS1 and revertant ovules in the endogenous contents of various other BRs, except for homocastasterone (homoCS), which was significantly higher at both pre-anthesis and anthesis in revertant ovules, and 6-deoxo-norcastasterone (6-deoxo-norCS), which was significantly elevated in BS1 ovules during pre-anthesis (Fig. 6A, B).

At pre-anthesis, 6-deoxo-norCS can be readily converted into two steps to the active BR forms, castasterone (CS) and brassinolide. These findings indicate that the higher concentrations of the bioactive brassinolide available in BS1 may arise from differences in the expression of BR-related biosynthetic and regulatory genes.

### Differential expression of BR biosynthetic and regulatory genes in BS1

**Fig. 7:**
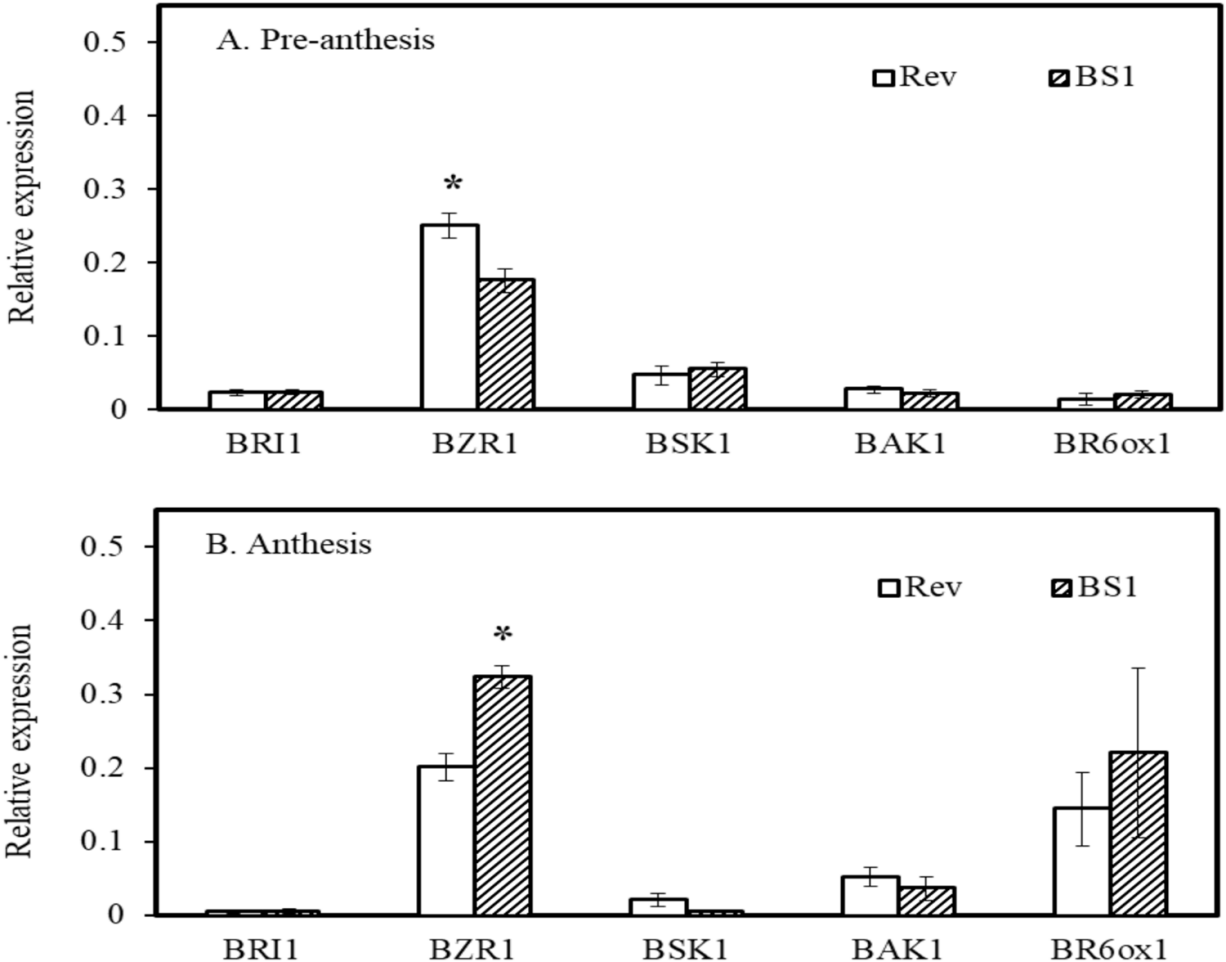
Expression levels of BRI1, BZR1, BSK1, BAK1, and BR6ox1 genes coding for enzymes involved in biosynthesis and signaling pathways of brassinosteroids in ovules of BS1 and revertant flower buds. Ovules were extracted from flower buds during pre-anthesis and at anthesis. RNA was prepared and levels of expression were determined by RT-qPCR. Data are presented as means ± SE (*n* = 4) and were analyzed using Student’s t test. Asterisk denotes statistically significant difference at *P* < 0.05

The expression of BR-related genes in BS1 and revertant ovules was determined by analyzing RNA extracted from the ovules at the pre-anthesis and anthesis stages. Subsequently, six libraries were constructed using an Illumina sequencing platform for RNA-Seq analysis (Supplementary Fig. S1). Further expression analysis through qPCR focused on key BR-related genes among the DEGs, namely, the BR receptors BRI1 and BAK1, the serine/threonine kinase BSK1 that acts as a positive regulator of BR signaling downstream of the receptor kinase BRI1, the transcription factor BZR1, and the biosynthetic gene C6 oxidase BR6ox1. The expression levels of the receptors BRI1 and BAK1 were similar at both pre-anthesis and anthesis in BS1 and revertant ovules, but the expression of BR6ox1 was higher, although not statistically different, in BS1 ovules at anthesis, probably contributing to elevated levels of active brassinolide in the ovules (Fig. 7A, B).

BZR1 expression revealed that it was significantly lower in BS1 ovules than in revertant ovules at the pre-anthesis stage and significantly higher at anthesis (Fig. 7A, B).

### Expression of genes encoding cell-wall-modifying enzymes is higher in BS1 than revertant ovules

The morphological variations observed in the ovules of BS1, i.e., enlarged ovules that develop to seed-like stony structure, require increased expression of cell-wall-modifying enzymes. Therefore, DEGs related to plant cell-wall-modifying enzymes were identified (Supplementary Table S1). Notably, it was found that various enzymes involved in cell-wall metabolism and modification exhibited significantly higher expression in BS1; these included xyloglucan xyloglucosyl transferase, pectinesterase, cellulose synthase-like protein, polygalacturonase, xyloglucan 6-xylosyltransferase, chitinase, endoglucanase, galacturan 1,4-a-galacturonidase, beta-galactosidase, and beta-glucosidase (Supplementary Table S1). Although the expression of these genes was not verified by PCR, it is evident that the induction of genes related to cell-wall modification occurred in BS1 ovules but not in revertant ovules. The marked elevation in expression of cell-wall-modifying enzymes in BS1 ovules may explain the observed increase in cell-wall thickness and the formation of stony seed-like structures.

## Discussion

### High levels of BRs correspond to alterations in the morphology of ovules, flowers, and stems in the BS1 mutant

The elevated levels of BRs in the BS1 mutant impact the anatomy of the ovules, flower buds, and stems. The high levels of BRs explain the elongation of organs, including the flower buds and stems as noted in previously (Figs. 1, 2, Wiess et al., 1993). Revertant fruits, bearing viable seeds, have the same shape as fruits of the progenitor cultivar'Ofer'due to the reduced levels of BRs compared to BS1. BS1 plants exhibit earlier flowering and fruit ripening, larger ovules, and fewer, smaller spines (formed by the differentiation of leaf primordia) on the peel vs. plants of the progenitor cultivar'Ofer'(Weiss et al. 1993). The fruit growth and accelerated ripening of BS1 vs. revertant fruits are probably induced by BRs (Fu et al. 2008; Tian et al. 2018), as has been previously observed in *A. thaliana* and cucumber (Fu et al. 2008; Li et al. 2024). Similar phenomena, such as early fruit development and the induction of parthenocarpic fruit, have previously been observed in cucumber following exogenous BR application (Fu et al. 2008).

At the cellular level, BRs stimulate cell elongation and expansion (González-García et al. 2011; Oh et al. 2020) by promoting the expression of genes encoding cell-wall-modifying enzymes and expansins, which are involved in remodeling of the cell wall (Park et al. 2010; Rao and Dixon 2017; Gutiérrez-Villamil et al. 2024). Transcriptomic analysis revealed significant up-regulation of cell-wall-related enzymes in BS1 (Supplementary Table S1), implying that the altered phenotypic traits observed in BS1 plants can be attributed to the influence of elevated levels of BRs on cellular processes, particularly those involved in cell-wall modification and expansion. BS1 mutant fruits exhibit larger ovules due to integument proliferation and thickening, which blocks their micropylar end, impeding pollen tube penetration and leading to fertilization failure. A similar enlarged ovule phenotype was observed in parthenocarpic tomato fruit due to proliferation of ovule integument cells (Gupta et al. 2021).

BRs function as essential regulators in reproductive growth and seed production; in *A. thaliana* and pea, they have been shown to govern male fertility, anther and pollen development, and seed size and shape (Nomura et al. 2007; Ye et al. 2011; Jiang et al. 2013; Li et al. 2024). BRs also play pivotal roles in flowering time and morphology in *A. thaliana* (Lima and Figueiredo 2024), with exogenous application of brassinolide promoting flowering and accelerating the transition from vegetative to reproductive growth stages (Li et al. 2024; Lima and Figueiredo 2024). In keeping with these findings, flowering in BS1 occurs about 2 weeks earlier than revertant flowering, and in accordance, BS1 fruits ripen earlier (Weiss et al. 1993).

### Induction of BZR1 expression and brassinolide production in mutant ovules of BS1

In the current study, transcriptomic and expression analyses of BS1 mutant ovules showed that BZR1 levels are up-regulated in parthenocarpic fruit in concert with enhanced brassinolide accumulation (Figs. 4, 5, 6 and 7). Compared to pre-anthesis levels, brassinolide concentrations during anthesis were significantly higher, and expression of BZR1 was also significantly enhanced during anthesis vs. pre-anthesis (Figs. 5, 7). Thus, the induction of parthenocarpic fruit is possibly triggered by BZR1, which coordinates the expression of the BR biosynthetic gene BR6ox1 and several cell-wall-related enzymes (Supplementary Table S1; Fig. 7). Although pre-anthesis and anthesis BS1 ovules express higher (but not statistically significant) BR6ox1 compared to revertant ovules, the difference is sufficient to induce the development of unfertilized ovules. In *A. thaliana*, BZR1 directly influences several genes involved in integument cell elongation and in seed development, including the regulation of seed size and shape (Jiang et al. 2013; Huang et al. 2013). BR mediates the development of the embryo and endosperm by regulating the expression of BZR1 target genes, thus influencing seed development (Jia et al. 2020). In the *bin2 A. thaliana* mutant, some ovules fail to develop into seeds due to defects in fertilization and zygote development (Huang et al. 2013). BR deficiency mutants display reduced seed numbers due to shorter pollen tubes, decreased fertilization efficiency, and reduced ovule initiation and development, thereby suppressing seed development through both maternal and paternal pathways (Huang et al. 2013). In BS1, unlike the *bin2 A. thaliana* mutant, the pollen tube elongates normally but cannot penetrate the blocked micropylar end of the ovule (Weiss et al. 1993). However, the parthenocarpic fruitlet continues to develop into a full-size fruit due to high levels of BRs and GAs. We note here that in contrast, male sterile fruitlets of the progenitor ‘Ofer’ containing unfertilized ovules failed to develop into full-size fruits (Weiss et al. 1993; Sitrit unpublished data).

### BRs–Gas interaction is critical for parthenocarpic fruit development in prickly pear

Fruit development in flowering plants is a complex process regulated by a network of molecular, hormonal, and environmental factors (Mesejo et al. 2016; Lima and Figueiredo 2024). Diverse hormonal signaling by auxins, cytokinins, abscisic acid, Gas, and BRs plays central roles in coordinating fruit set and seed development (Domagalska et al. 2010; Depuydt and Hardtke 2011; Li et al. 2024). In *A. thaliana*, for example, four genes associated with GA regulation are directly influenced by BZR1 (Wang et al. 2012), constituting an additional layer of interaction between the BR and GA pathways, which share common biological functions in fruit set, seed regulation, and germination. In the complex interaction between the two groups of hormones, GA stabilizes BZR1 and BZR1 induces the degradation of the GA repressor DELLA proteins, while DELLA proteins inhibit BR signaling (Wang et al. 2012). For example, the intricate interplay between BZR1 and DELLA proteins was found to be pivotal in coordinating seed development in rice (Tong et al. 2014). In keeping with the above, we found that BS1 ovules contain higher levels of the active gibberellins GA1, GA3, and GA4 and enhanced expression of the genes involved in GA synthesis (GA20ox) and perception (GID1). In contrast, revertant ovules had significantly higher expression of the catabolic gene GA2ox (article in preparation).

Our study, using the BS1 mutant as a model system, provides valuable insights into the regulatory mechanisms governing parthenocarpic fruit development in prickly pear. In light of our findings, we propose that the roles of BRs in parthenocarpic BS1 fruit development are controlled by the BZR1 regulator. The higher levels of endogenous BRs, especially brassinolide, in BS1 vs. revertant ovules suggest that, in the absence of fertilization, brassinolide supports the development of parthenocarpic fruits in the BS1 mutant. The results of this study imply that a complex mechanism involving GAs–BRs interactions induce parthenocarpy in prickly pear fruit. Overall, this study advances our understanding of the hormonal regulation of fruit and seed development in prickly pear and sets the stage for future research aimed at developing seedless fruit with enhanced consumer appeal through targeted manipulation of BR signaling pathways.

## Supplementary Information

Below is the link to the electronic supplementary material.

Supplementary file 1 (DOCX 427 KB)

## Data Availability

The data supporting the findings are accessible both within the paper and in the supplementary materials available online. Data will be shared on request to the corresponding author.
