# Supplementary material for: Brassinolide and BZR1 are up-regulated in a parthenocarpic mutant of prickly pear
Source: Plant Cell Rep. 2025 May 23;44(6):131. doi: 10.1007/s00299-025-03514-w (PMC12102005; doi:10.1007/s00299-025-03514-w)
Supplement: Supplementary file 1 — (DOCX 427 KB) [file 299_2025_3514_MOESM1_ESM.docx]

**Supplementary data**

**Table S1.** Expression of genes coding for enzymes modifying cell-wall polysaccharides in BS1 ovules compared to expression in revertant ovules. RNA was extracted from ovules, and libraries were prepared. All genes of cell-wall-modifying enzymes (n=3) in BS1 ovules exhibited significant expression (p<0.05) compared to revertant ovules and are expressed as log2 fold change.

| Enzyme | Gene name/ transcript ID | Log2 fold change | Differentially expressed genes in BS1 |
| --- | --- | --- | --- |
| Xyloglucan xyloglucosyl transferase [EC:2.4.1.207] | *Ofi_XT1*  *Ofi_XT2*  *Ofi_XT3*  *Ofi_XT4*  *Ofi_XT5*  *Ofi_XT6*  *Ofi_XT7*  *Ofi_XT8*  *Ofi_XT9*  *Ofi_XT10*  *Ofi_XT11*  *Ofi_XT12* | +2.24  +2.04  +1.79  +1.28  +5.70  +0.98  +1.51  +2.26  +1.78  +2.51  +2.89  +1.78 | Up regulation |
| Pectinesterase [EC:3.1.1.11] | *Ofi_PE1*  *Ofi_PE2*  *Ofi_PE3*  *Ofi_PE4* | +1.05  +0.89  +1.91  +0.67 | Up regulation |
| Cellulose synthase-like protein [EC:2.4.1.-] | *Ofi_* *CSLD1* | +1.89 | Up regulation |
| Polygalacturonase [EC:3.2.1.15] | *Ofi_PG1* | +10.99 | Up regulation |
| Xyloglucan 6-xylosyltransferase [EC:2.4.2.39] | *Ofi_XXT1* | +1.28 | Up regulation |
| Chitinase [EC:3.2.1.14] | *Ofi_CHIT1*  *Ofi_CHIT2*  *Ofi_CHIT3*  *Ofi_ CHIT4* | +3.02  +1.62  +3.27  +1.37 | Up regulation |
| Endoglucanase [EC:3.2.1.4] | *Ofi_EG1*  *Ofi_EG2*  *Ofi_EG3*  *Ofi_EG4* | -2.11  -1.54  -1.58  -1.49 | Down regulation |
| Galacturan 1,4-alpha-galacturonidase [EC:3.2.1.67] | *Ofi_GAG1*  *Ofi_GAG2* | -6.02  +0.80 | Up/down regulation |
| β-galactosidase [EC:3.2.1.23] | *Ofi_BGA1*  *Ofi_BGA2*  *Ofi_BGA3*  *Ofi_BGA4* | +0.63  +2.11  +1.02  +0.79 | Up regulation |
| Beta-glucosidase [EC:3.2.1.21] | *Ofi_BGL1*  *Ofi_BGL2*  *Ofi_BGL3*  *Ofi_BGL4*  *Ofi_BGL5*  *Ofi_BGL6*  *Ofi_BGL7*  *Ofi_BGL8* | +1.07  +2.93  +2.86  +1.10  +1.78  +0.94  +0.85  +3.99 | Up regulation |

**
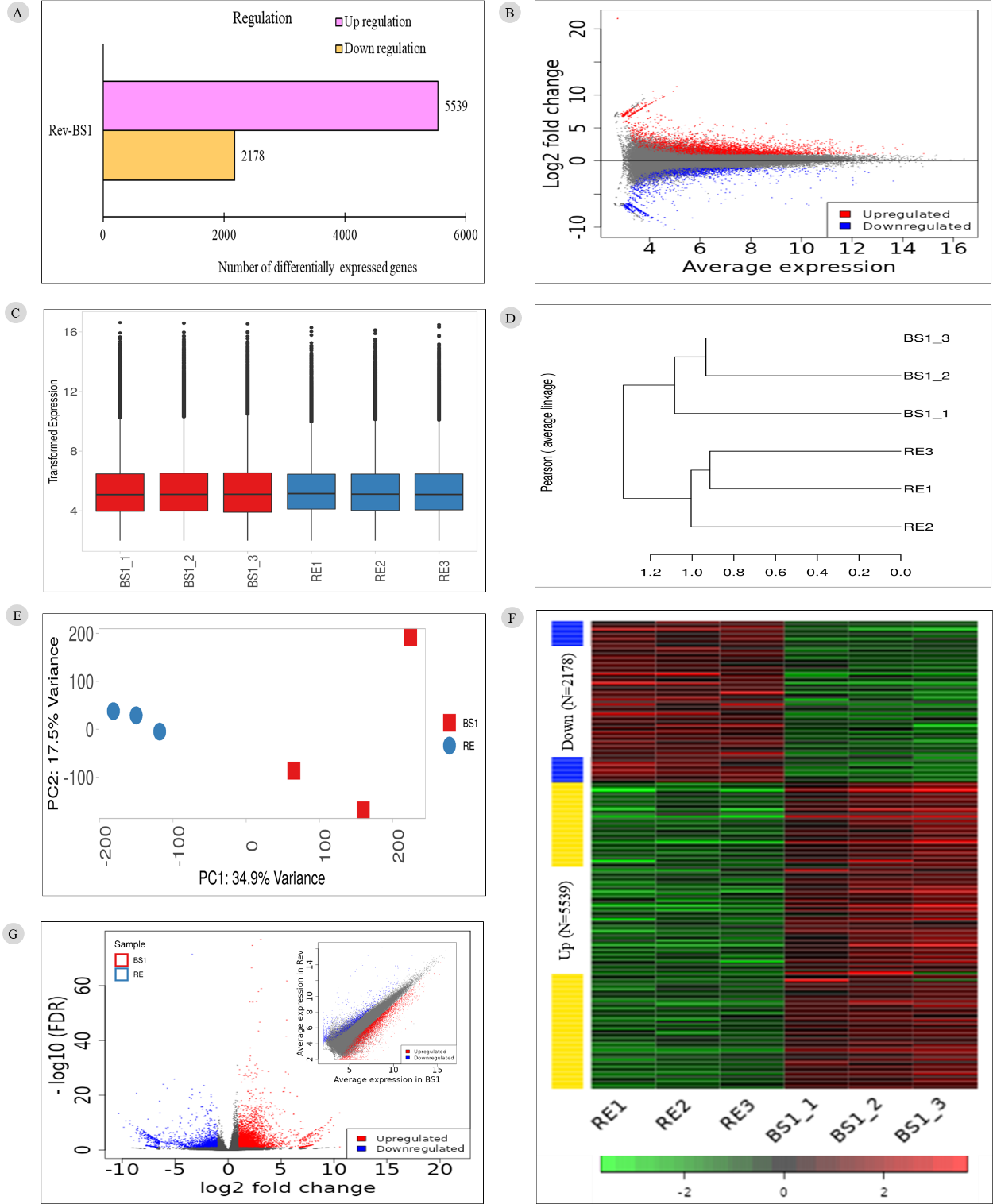
**

**Fig. S1.** Transcriptome analysis of BS1 and revertant (Rev). (A) Differential expression of genes in BS1 and Rev. (B) Scatter plot of transformed expression in BS1 and Rev, showing a strong positive relationship. (C) Box plot showing a comparative analysis of data dispersion within and between the BS1 and revertant groups for transformed data distribution. (D) Phylogenetic tree showing the genetic relationships between BS1 and Rev groups. (E) PCA analysis of BS1 versus revertant. (F) Hierarchical clustering. (G) Volcano plot of ddifferentially expressed genes (DEGs) (Inset: MA plot of DEGs).
